# Uptake and safety profile of anti-amyloid immunotherapies in routine clinical practice

**DOI:** 10.1177/25424823261483526

**Published:** 2026-09-30

**Authors:** Jay B. Lusk, Kate Vinita Fitch, Kim G. Johnson, Andy Liu, Jennifer L. Lund, Laine E. Thomas, Ryan McDevitt, Anqi Zhao, Heather E. Whitson, Richard O'Brien, Shannon Aymes, Bradley G. Hammill, Brian Mac Grory, Fan Li, Emily C. O'Brien

**Affiliations:** 1 Departments of Neurology and Internal Medicine, University of Texas Southwestern Medical Center; 2 Department of Family Medicine, University of North Carolina, Chapel Hill, NC, USA; 3 Observational Research Building Interdisciplinary Therapeutic Advances (ORBIT) Hub, Duke University, Durham, NC, USA; 4 Department of Neurology, 213852 Duke University, Durham, NC, USA; 5 Department of Health Economics, Systems, and Policy, Peter J. O'Donnel l Jr School of Public Health, University of Texas Southwestern Medical Center, Dallas, TX, USA; 6 Department of Epidemiology, Gillings School of Global Public Health, 2331 University of North Carolina-Chapel Hill, Chapel Hill, NC, USA; 7 Department of Psychiatry and Behavioral Science, 213852 Duke University, Durham, NC, USA; 8 Department of Statistical Science, 213852 Duke University, Durham, NC, USA; 9 Clinical Research Institute, 213852 Duke University, Durham, NC, USA; 10 Fuqua School of Business, 213852 Duke University, Durham, NC, USA; 11 Olin School of Business, 7548 Washington University, St Louis, MO, USA; 12 School of Public Health, 7548 Washington University, St Louis, MO, USA; 13 Department of Medicine, 3065 Duke University, Durham, NC, USA; 14 Geriatrics Research Education and Clinical Center, Durham VA Medical Center, Durham, NC, USA; 15 Department of Population Health Sciences, 3065 Duke University, Durham, NC, USA

**Keywords:** Alzheimer's disease, amyloid targeting therapies, donanemab, lecanemab, safety, target trial emulation

## Abstract

**Background:**

Anti-amyloid immunotherapies are approved by the United States Food and Drug Administration for the treatment of Alzheimer's disease; however, the adoption and safety profile of these medications in routine clinical practice have not been described.

**Objective:**

To describe the adoption and safety profile of anti-amyloid immunotherapies in routine clinical practice.

**Methods:**

This was a retrospective observational cohort study using target trial emulation methods. The data source was nationwide electronic health record data from Epic Cosmos. The primary exposure was the first prescription of anti-amyloid immunotherapy versus the active comparator of oral dementia therapy (acetylcholinesterase inhibitor (AChEI) or memantine, selected to minimize bias by baseline health status and disease severity). The primary end point was time to first nontraumatic intracranial hemorrhage (ICH). Propensity score overlap weighting was used to balance baseline demographic and clinical characteristics across treatment groups.

**Results:**

Between July 1, 2023 and January 1, 2025, 2616 patients (median age 74.8 years [IQR 69.8–78.8]; 53.9% female) were prescribed anti-amyloid immunotherapy (with or without AChEI/memantine), and 1,065,192 patients (median age 79.98 [IQR 73.6–85.6], 57.9% female) were prescribed AChEI/memantine alone. In total, 401 patients prescribed anti-amyloid immunotherapy and 274,470 patients prescribed AChEI/memantine were assessed for safety endpoints. Compared with AChEI/memantine, the analysis did not detect a statistically significant association between prescription of anti-amyloid immunotherapy and ICH (owHR 0.73 [95% CI 0.10–5.12]).

**Conclusions:**

The study did not detect a statistically significant association between prescription of anti-amyloid immunotherapy and ICH. The small treated cohort and low event count yielded imprecise estimates; these findings do not confirm safety and cannot exclude a clinically important increase in risk.

## Introduction

Pivotal trials demonstrated the efficacy of monoclonal antibody therapy targeting amyloid-β (Aβ) in slowing the clinical progression of Alzheimer's disease, leading to accelerated approval of aducanumab in June 2021, followed by accelerated approval of lecanemab in January 2023 (with full approval in July 2023), then full approval of donanemab in July 2024.^1–4^ The balance of benefits and harms of these therapies has been a subject of intense debate, particularly due to the risk of amyloid-related imaging abnormalities (ARIA).^5–9^ Although recent studies have estimated low treatment eligibility (approximately 5–8% of patients with mild cognitive impairment or mild dementia),^10–13^ the real-world uptake and safety profile of these medications in the United States are not yet known.

It is important to understand the risks of harm associated with this therapy in routine clinical practice.^14–16^ Therefore, the objectives of this study were twofold: 1) to describe the uptake of anti-amyloid immunotherapy in routine clinical practice and 2) to determine if the prescription of anti-amyloid immunotherapy was associated with an increased risk of key safety end points compared to acetylcholinesterase inhibitors (AChEI) or memantine.

## Methods

### Study design

This was a retrospective observational cohort study which was designed and reported according to a prespecified statistical analysis plan (Supplemental Material).

### Data source

Data used in this study were obtained from Epic Cosmos, a data set created in collaboration with a community of Epic health systems representing more than 300 million patient records from more than 1748 hospitals and 40,700 clinics from all 50 states, the District of Columbia, Lebanon, and Saudi Arabia. Data available include structured (billing and procedural codes) and semi-structured data (patient demographics, diagnoses, procedures, physical measurements and laboratory measurements) which are harmonized across sites.

### Ethical approval and reporting

The study was determined exempt from institutional review board review by the University of North Carolina Chapel Hill institutional review board (review number 459493). The study was reported according to the RECORD and TARGET statements (Supplemental Material).

### Study population

The study population included all patients who received their first prescription for an anti-amyloid immunotherapy (lecanemab or donanemab) or an AChEI (donepezil, rivastigmine, or galantamine) or NMDA receptor antagonist (memantine) between July 1, 2023 and January 1, 2025. The comparator population of patients receiving an AChEI was selected to allow for alignment of index date and to contextualize the background risk of adverse events unrelated to anti-amyloid immunotherapy. No participants were excluded from the study when evaluating uptake of anti-amyloid immunotherapy. For the safety outcome analysis, a sub-cohort was created by applying a set of exclusion criteria based on a two-year look back period from the initial prescription date. Exclusions included a history of ischemic or hemorrhagic stroke, atrial fibrillation/flutter, cardiac valvular surgery, prescription of warfarin or any non-vitamin K oral anticoagulant, deep venous thrombosis, a hospice/palliative services in the lookback period, or being aged 90 years or older the index date. The derivation from 2616 patients ever prescribed anti-amyloid immunotherapy to 401 patients in the anti-amyloid immunotherapy safety cohort reflected treatment sequencing and the prespecified exclusions displayed in Figure 1.

**Figure 1.**
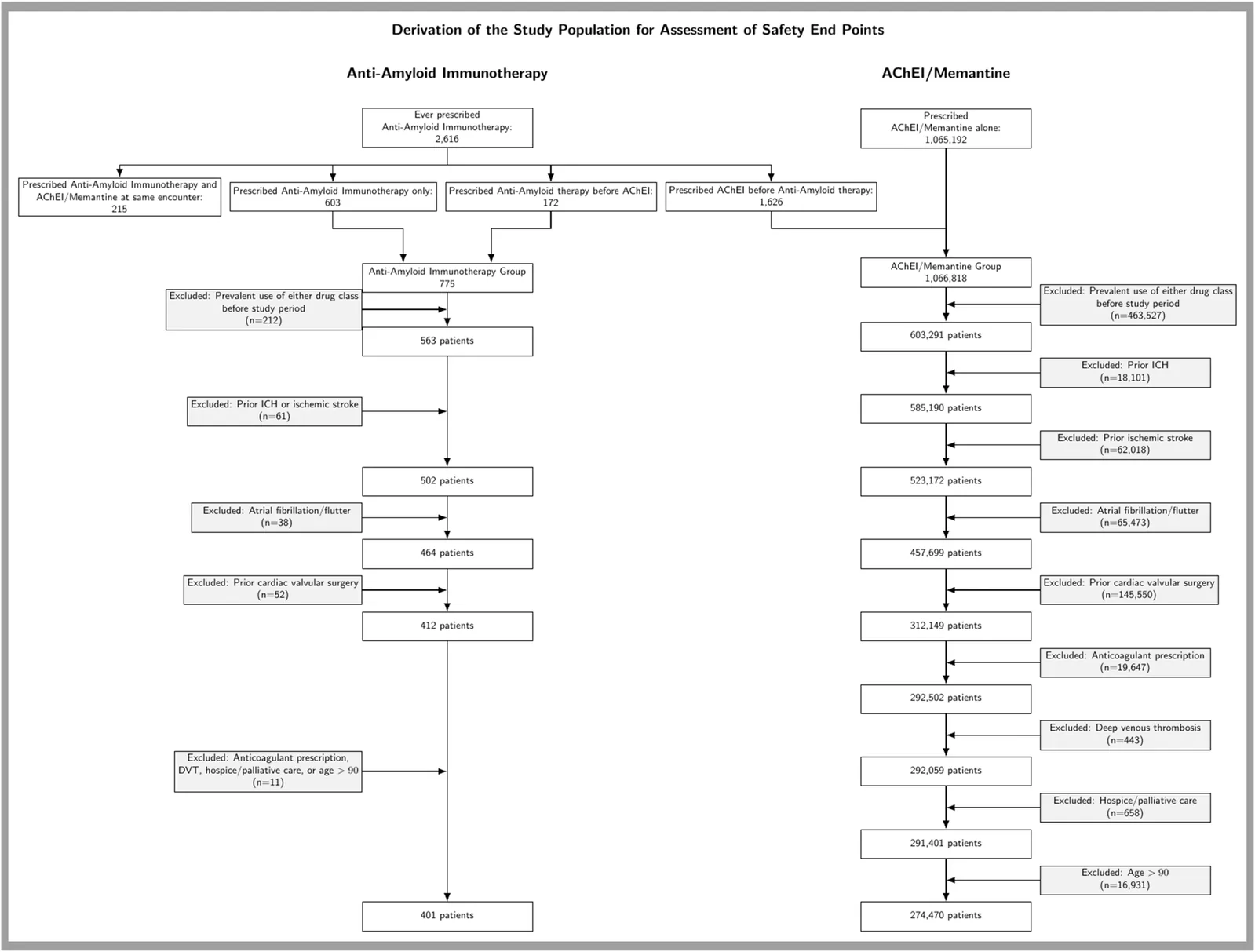
Derivation of the patient population used for safety analysis.

### Exposures

The primary exposure was prescription of anti-amyloid immunotherapy, either lecanemab or donanemab, between July 1, 2023 and January 1, 2025. The index date was defined as the first prescription date for either anti-amyloid immunotherapy or an AChEI/memantine. Patients were assigned to one of two groups: those receiving anti-amyloid immunotherapy or a comparator group receiving AChEI/memantine. In cases of combination therapy, patients were assigned to the group for the medication that was prescribed first. A pre-specified sensitivity analysis was performed using a clone-censor-weight approach to account for the fact that many patients were expected to use both therapies.^17^ Aducanumab was excluded a priori because the prespecified exposure definition focused on lecanemab and donanemab, the agents entering routine clinical use during the study window, while aducanumab had a materially different and declining commercial availability and implementation context. Consequently, the class-level findings apply to lecanemab and donanemab and should not be extrapolated to aducanumab or to all anti-amyloid agents. Safety analyses were conducted at the drug-class level rather than separately for lecanemab and donanemab because treatment-specific event counts were sparse.

### End points

The primary end point was first diagnosis of nontraumatic intracranial hemorrhage (ICD-10-CM I61.* or ICD-10-CM I62.*), encompassing nontraumatic intracerebral hemorrhage (I61.*) and other nontraumatic intracranial hemorrhage (I62.*). This endpoint was chosen because while ARIA do not have a specific ICD-10 code, a severe manifestation of ARIA (such as ARIA-H/hemorrhage) is highly likely to be captured as a nontraumatic intracranial hemorrhage. The code-based endpoint cannot distinguish ARIA-H from spontaneous intracranial hemorrhage (ICH) and does not capture ARIA-E without hemorrhage. Thus, ICH was analyzed as a pragmatic surrogate for severe hemorrhagic manifestations and not as a measure of overall ARIA incidence. Secondary end points included first myocardial infarction, ischemic stroke, and composite of myocardial infarction, ischemic stroke, and intracranial hemorrhage, diagnosis with diarrheal illness or vomiting, and diagnosis of headache. Additional end points included first emergency department visit or hospitalization as well as count of emergency department visits and hospitalizations over 1 year after first prescription. A negative control (falsification) endpoint of cataract diagnosis was used to assess residual confounding, as it is a common age-related condition that requires health system engagement but is not biologically linked to either treatment.

Exploratory safety end points based on immune-related adverse effects seen in other immunotherapies, including diagnosis of lower or upper respiratory tract infection, myocarditis or pericarditis, acute liver injury, interstitial lung disease, severe skin reactions, hypothyroidism or hyperthyroidism, and hyperosmolar hyperglycemic state or diabetic ketoacidosis. The specific codes for all endpoints are detailed in the Supplemental Material.

### Statistical analysis

Patient characteristics were summarized using counts and percentages for categorical variables and means and standard deviations for continuous variables and standardized mean differences (SMDs) were used to compare characteristics between groups. Covariate balance was assessed before and after overlap weighting; pre-weighting SMDs are reported in Table 2 and post-weighting SMDs in Supplemental Table 2. Propensity score overlap weighting was used to account for measured confounding because it effectively handles extreme propensity scores and ensures covariate balance across groups.^18,19^ Variables that were collinear or exhibited low variance were omitted as detailed in the Supplemental Material. The propensity score model included a set of variables defined using a two-year look-back period, including:

- Demographics: Age, sex, race, and ethnicity
- Social determinants of health: Social vulnerability index, rural urban commuting area (RUCA) classification, marital status, and state/province.
- Physical measurements: Systolic blood pressure, diastolic blood pressure, body mass index, oxygen saturation, and heart rate
- Laboratory measurements: Hemoglobin, hematocrit, white blood cell count, platelet count, aspartate aminotransferase, alanine aminotransferase, serum creatinine, sodium, potassium, glycated hemoglobin, low density lipoprotein cholesterol, high density lipoprotein cholesterol, serum triglycerides
- Prior procedures: Coronary artery bypass grafting, carotid endarterectomy, cardiac ablation, pacemaker placement, and percutaneous coronary intervention, as well as the procedural components of the Claims-based Frailty Index (CFI).^20^
- Prior diagnoses: Individual components of the Elixhauser comorbidity index, as well as the individual components of the CFI and common medical conditions affecting patients with cognitive impairment, as detailed in the Supplemental Material.^21,22^
- Current medications: Antihypertensive, diuretic, antithrombotic, lipid lowering, antiglycemic, antidepressant, anxiolytic, antipsychotic, endocrine, respiratory, gastrointestinal, opioid, and antibiotic medications, as detailed in the Supplemental Material.
- Prior healthcare utilization: Number of outpatient encounters, emergency department encounters, hospitalizations, and number of visits to neurologists, geriatricians, psychiatrists, primary care doctors, and cardiologists.

Time to event endpoints were analyzed using Cox proportional hazard models, with patients censored at death, end of data availability, or at one year after the index date; thus, the maximum follow-up was one year. The primary analysis followed initial treatment assignment, and treatment switching was addressed in a prespecified clone-censor-weight sensitivity analysis. For count endpoints, Quasi-Poisson regression was used to account for overdispersion, and the *survey* package was used to implement overlap weights for adjusted models.^23^ Missing data was imputed using random forest-based imputation, given the large number of adjustment variables of different types.^24^ Analyses were performed in Microsoft SQL Server and R with RStudio. The threshold for two-sided tests was set at alpha = 0.05; no adjustment was made for multiple comparisons. E-values were calculated for primary end points to assess the potential impact of unmeasured confounding on study results.^25^

## Results

### Characteristics of patients ever receiving anti-amyloid immunotherapy versus AChEI therapy alone

In total, 2616 patients (median age 74.8, IQR 69.8–78.8, 53.9% female) received anti-amyloid immunotherapy (with or without AChEI/memantine), and 1,065,192 patients (median age 79.98, IQR 73.6–85.6, 57.9% female) received AChEI/memantine alone over the study period. Compared with patients prescribed AChEI/memantine, those prescribed anti-amyloid immunotherapy were younger (mean age 74 versus 79, SMD 0.506), less likely to be female (57.9% versus 53.9%, SMD 0.081), less likely to be Hispanic/Latino (2.2% versus 6.7%, SMD 0.227), more likely to be White (86.6% versus 74.1%, SMD 0.317), and less likely to be Asian (1.1% versus 2.1%, SMD 0.083) or Black (1.8% versus 9.9%, SMD 0.353). Those prescribed anti-amyloid immunotherapy were also more likely to be from urban areas (89.1% versus 84.2%, SMD 0.146), and less likely to be from suburban (6.5% versus 9.4%, SMD 0.108) or rural areas (3.0% versus 4.8%, SMD 0.093), more likely to reside in ZIP codes with higher socioeconomic status (mean SVI percentile 46 versus 56, SMD 0.392), and more likely to be married (80.9% versus 50.9%, SMD 0.666) instead of divorced, single, or widowed (Table 1).

**Table 1.** Characteristics of patients who were ever prescribed anti-amyloid immunotherapy versus those prescribed only acetylcholinesterase inhibitors or memantine.

Variable	AChEI/memantine alone (n = 1,065,192)	Anti-amyloid immunotherapy (n = 2616)	SMD
*Demographics*			
Age	78.55	73.92	0.506
Sex			
Female, n (%)	616,811 (57.9%)	1411 (53.9%)	0.08
Male, n (%)	448,271 (42.1%)	1205 (46.1%)	0.08
Race			
American Indian or Alaska Native, n (%)	2406 (0.2%)	<11 (<0.4%)	0.067
Asian, n (%)	22,406 (2.1%)	28 (1.1%)	0.083
Black or African American, n (%)	105,482 (9.9%)	46 (1.8%)	0.353
More than one Race, n (%)	96,274 (9.0%)	232 (8.9%)	0.006
Native Hawaiian or Other Pacific Islander, n (%)	887 (0.1%)	<11 (<0.4%)	0.018
Other Race, n (%)	26,852 (2.5%)	17 (0.6%)	0.15
White, n (%)	789,622 (74.1%)	2265 (86.6%)	0.317
Ethnicity			
Hispanic or Latino, n (%)	71,081 (6.7%)	57 (2.2%)	0.227
Not Hispanic or Latino, n (%)	942,387 (88.5%)	2448 (93.6%)	0.227
Marital status			
Divorced or Legally Separated, n (%)	98,631 (9.3%)	120 (4.6%)	0.187
Married, n (%)	541,683 (50.9%)	2117 (80.9%)	0.666
Other, n (%)	32,543 (3.1%)	57 (2.2%)	0.056
Unmarried, n (%)	126,863 (11.9%)	104 (4.0%)	0.299
Widowed, n (%)	256,245 (24.1%)	210 (8.0%)	0.452
Rural-urban commuting area (RUCA) group			
Rural, n (%)	50,929 (4.8%)	79 (3.0%)	0.093
Suburban, n (%)	99,634 (9.4%)	170 (6.5%)	0.108
Urban, n (%)	896,642 (84.2%)	2332 (89.1%)	0.146
U.S. Census division			
East North Central, n (%)	204,904 (19.2%)	387 (14.8%)	0.12
East South Central, n (%)	70,929 (6.7%)	251 (9.6%)	0.107
Middle Atlantic, n (%)	132,402 (12.4%)	327 (12.5%)	0.001
Mountain, n (%)	49,791 (4.7%)	98 (3.7%)	0.047
New England, n (%)	54,687 (5.1%)	233 (8.9%)	0.148
Pacific, n (%)	72,721 (6.8%)	110 (4.2%)	0.116
South Atlantic, n (%)	268,872 (25.2%)	629 (24.0%)	0.03
West North Central, n (%)	63,648 (6.0%)	148 (5.7%)	0.014
West South Central, n (%)	135,417 (12.7%)	411 (15.7%)	0.085
Social Vulnerability Index (percentile, 2020, by ZIP)	0.56	0.46	0.392
*Laboratory variables*			
High-density lipoprotein, mg/dL	54.56	61.08	0.367
Low-density lipoprotein, mg/dL	87.72	93.96	0.171
Serum Triglycerides, mg/dL	122.87	107.98	0.217
Hematocrit (%)	38.86	41.16	0.486
Hemoglobin, mg/dL	12.73	13.61	0.531
White blood cell count, cells/uL	8.12	6.84	0.389
Platelet count, cells/uL	232.52	233.9	0.019
Potassium, mEq/L	4.18	4.26	0.171
Sodium, mEq/L	139.25	139.64	0.115
Serum Creatinine, mg/dL	1.14	0.95	0.321
ALT, u/L	22.59	21.35	0.046
AST, u/L	27.33	23.93	0.114
HbA1c (%)	6.46	6.13	0.272
*Medical history*			
Myocardial infarction, n (%)	62,516 (5.9%)	52 (2.0%)	0.201
Congestive heart failure, n (%)	137,393 (12.9%)	79 (3.0%)	0.371
Peripheral vascular disease, n (%)	116,374 (10.9%)	156 (6.0%)	0.179
Cerebrovascular disease, n (%)	165,915 (15.6%)	330 (12.6%)	0.085
Chronic obstructive pulmonary disease (COPD), n (%)	158,510 (14.9%)	152 (5.8%)	0.301
Rheumatologic disease, n (%)	31,478 (3.0%)	70 (2.7%)	0.017
Peptic ulcer disease, n (%)	9820 (0.9%)	<11 (<0.4%)	0.067
Mild liver disease, n (%)	35,398 (3.3%)	45 (1.7%)	0.102
Diabetes mellitus (no complications), n (%)	224,053 (21.0%)	248 (9.5%)	0.326
Diabetes mellitus with complications, n (%)	138,267 (13.0%)	138 (5.3%)	0.27
Paralysis, n (%)	16,615 (1.6%)	<11 (<0.4%)	0.136
Chronic kidney disease, n (%)	197,198 (18.5%)	161 (6.2%)	0.383
Any malignancy (excluding skin), n (%)	95,354 (9.0%)	188 (7.2%)	0.065
Moderate to severe liver disease, n (%)	3417 (0.3%)	<11 (<0.4%)	0.067
Metastatic solid tumor, n (%)	19,584 (1.8%)	12 (0.5%)	0.13
HIV/AIDS, n (%)	1395 (0.1%)	<11 (<0.4%)	0.005
Cardiac arrhythmia, n (%)	285,298 (26.8%)	350 (13.4%)	0.339
Depression, n (%)	230,530 (21.6%)	502 (19.2%)	0.061
Drug abuse, n (%)	19,686 (1.8%)	17 (0.6%)	0.108
Psychoses, n (%)	35,725 (3.4%)	91 (3.5%)	0.007
Deep venous thrombosis, n (%)	4537 (0.4%)	<11 (<0.4%)	0.092
Valvular heart disease, n (%)	103,159 (9.7%)	166 (6.3%)	0.123
Atrial fibrillation, n (%)	158,828 (14.9%)	134 (5.1%)	0.33
Atrial flutter, n (%)	17,501 (1.6%)	17 (0.6%)	0.093
Alcohol use disorder, n (%)	16,430 (1.5%)	28 (1.1%)	0.042
Anxiety disorders, n (%)	192,159 (18.0%)	498 (19.0%)	0.026
Chronic pain, n (%)	286,131 (26.9%)	546 (20.9%)	0.141
Epilepsy, n (%)	36,548 (3.4%)	28 (1.1%)	0.16
Fall-related injury, n (%)	200,038 (18.8%)	287 (11.0%)	0.221
Malnutrition, n (%)	92,038 (8.6%)	93 (3.6%)	0.214
Migraine, n (%)	28,430 (2.7%)	76 (2.9%)	0.014
Osteoarthritis, n (%)	154,399 (14.5%)	318 (12.2%)	0.069
Peripheral artery disease, n (%)	111,164 (10.4%)	136 (5.2%)	0.196
Pneumonia, n (%)	55,140 (5.2%)	23 (0.9%)	0.253
Urinary tract infection, n (%)	113,911 (10.7%)	94 (3.6%)	0.278
*Procedures*			
Coronary artery bypass grafting (CABG), n (%)	434 (0.0%)	<11 (<0.4%)	0.033
Electrical cardioversion, n (%)	2066 (0.2%)	<11 (<0.4%)	0.031
Pacemaker, n (%)	16,563 (1.6%)	24 (0.9%)	0.088
Defibrillator, n (%)	4364 (0.4%)	<11 (<0.4%)	0.084
Implantable cardiac monitor, n (%)	4104 (0.4%)	14 (0.5%)	0.01
Cardiac valve surgery, n (%)	1214 (0.1%)	<11 (<0.4%)	0.056
Percutaneous coronary intervention (stent), n (%)	3292 (0.3%)	<11 (<0.4%)	0.028
Peripheral arterial stent, n (%)	1411 (0.1%)	<11 (<0.4%)	0.026
*Prescription drug exposures*			
Angiotensin receptor blocker (ARB), n (%)	226,061 (21.2%)	510 (19.5%)	0.043
ACE inhibitor, n (%)	200,477 (18.8%)	363 (13.9%)	0.134
Calcium channel blocker, n (%)	293,988 (27.6%)	460 (17.6%)	0.241
Beta-blocker, n (%)	388,118 (36.4%)	598 (22.9%)	0.301
Potassium-sparing diuretic, n (%)	51,517 (4.8%)	72 (2.8%)	0.109
Thiazide diuretic, n (%)	134,339 (12.6%)	272 (10.4%)	0.069
Loop diuretic, n (%)	175,578 (16.5%)	125 (4.8%)	0.387
Alpha-1 antagonist, n (%)	21,559 (2.0%)	25 (1.0%)	0.088
Vasodilator, n (%)	154,544 (14.5%)	170 (6.5%)	0.264
Anticoagulant, n (%)	160,447 (15.1%)	114 (4.4%)	0.368
Antiplatelets, n (%)	288,794 (27.1%)	460 (17.6%)	0.23
Statin, n (%)	559,015 (52.5%)	1339 (51.2%)	0.026
Fibrates, n (%)	20,629 (1.9%)	49 (1.9%)	0.005
Bile acid sequestrant, n (%)	15,342 (1.4%)	32 (1.2%)	0.019
PCSK9 inhibitor, n (%)	6502 (0.6%)	49 (1.9%)	0.114
Ezetimibe, n (%)	38,715 (3.6%)	144 (5.5%)	0.09
Icosapent ethyl, n (%)	5463 (0.5%)	14 (0.5%)	0.003
Metformin, n (%)	123,337 (11.6%)	219 (8.4%)	0.107
Insulin, n (%)	160,801 (15.1%)	142 (5.4%)	0.323
GLP-1 receptor Agonists, n (%)	41,037 (3.9%)	92 (3.5%)	0.018
SGLT-2 Inhibitors, n (%)	52,819 (5.0%)	98 (3.7%)	0.059
Sulfonylureas, n (%)	49,617 (4.7%)	58 (2.2%)	0.134
DDP-IV Inhibitors, n (%)	31,281 (2.9%)	33 (1.3%)	0.117
Thiazolidinediones, n (%)	11,733 (1.1%)	22 (0.8%)	0.027
SSRI, n (%)	300,844 (28.2%)	816 (31.2%)	0.065
SNRI, n (%)	103,454 (9.7%)	267 (10.2%)	0.017
Tricyclic antidepressant (TCA), n (%)	37,893 (3.6%)	69 (2.6%)	0.053
Monoamine oxidase inhibitor, n (%)	1713 (0.2%)	<11 (<0.4%)	0.025
Buspirone, n (%)	47,476 (4.5%)	121 (4.6%)	0.008
Benzodiazepines, n (%)	295,224 (27.7%)	624 (23.9%)	0.088
Trazodone, n (%)	122,810 (11.5%)	220 (8.4%)	0.104
Bupropion, n (%)	54,877 (5.2%)	196 (7.5%)	0.096
Mirtazapine, n (%)	76,145 (7.1%)	76 (2.9%)	0.195
Gaba Analogs, n (%)	197,340 (18.5%)	306 (11.7%)	0.192
Typical Antipsychotics, n (%)	50,468 (4.7%)	42 (1.6%)	0.179
Second-generation antipsychotic, n (%)	168,863 (15.9%)	106 (4.1%)	0.402
Thyroid hormone replacement, n (%)	181,989 (17.1%)	399 (15.3%)	0.05
Bisphosphonate, n (%)	52,874 (5.0%)	153 (5.8%)	0.039
Short-acting β-agonist (SABA), n (%)	231,652 (21.7%)	298 (11.4%)	0.281
Short-acting muscarinic antagonist (SAMA), n (%)	127,479 (12.0%)	147 (5.6%)	0.226
Long-acting β-agonist (LABA), n (%)	78,594 (7.4%)	108 (4.1%)	0.14
Long-acting muscarinic antagonist (LAMA), n (%)	82,496 (7.7%)	149 (5.7%)	0.082
Inhaled corticosteroid, n (%)	158,163 (14.8%)	364 (13.9%)	0.027
Proton pump inhibitor, n (%)	318,146 (29.9%)	530 (20.3%)	0.223
H2 receptor blocker, n (%)	144,946 (13.6%)	286 (10.9%)	0.082
Opioids, n (%)	408,407 (38.3%)	768 (29.4%)	0.191
Penicillin (±beta-lactamase inhibitor), n (%)	140,611 (13.2%)	313 (12.0%)	0.037
First Generation Cephalosporins, n (%)	194,752 (18.3%)	325 (12.4%)	0.163
2nd-generation cephalosporin, n (%)	28,700 (2.7%)	45 (1.7%)	0.066
3rd-generation cephalosporin, n (%)	96,408 (9.1%)	107 (4.1%)	0.201
Fourth Generation Cephalosporins, n (%)	12,403 (1.2%)	<11 (<0.4%)	0.125
Fifth Generation Cephalosporins, n (%)	22 (0.0%)	<11 (<0.4%)	0.006
Carbapenem, n (%)	3909 (0.4%)	<11 (<0.4%)	0.042
Monobactam, n (%)	412 (0.0%)	<11 (<0.4%)	0.028
Macrolide, n (%)	96,750 (9.1%)	221 (8.4%)	0.022
Fluoroquinolones, n (%)	129,300 (12.1%)	234 (8.9%)	0.104
Tetracycline, n (%)	106,518 (10.0%)	231 (8.8%)	0.04
Aminoglycoside, n (%)	19,047 (1.8%)	60 (2.3%)	0.036
Sulfonamide, n (%)	6918 (0.6%)	<11 (<0.4%)	0.05
Lincosamide, n (%)	32,640 (3.1%)	72 (2.8%)	0.019
Glycopeptide, n (%)	52,285 (4.9%)	63 (2.4%)	0.133
Oxazolidinone, n (%)	5570 (0.5%)	<11 (<0.4%)	0.064
Nitroimidazole, n (%)	44,536 (4.2%)	89 (3.4%)	0.041
*Healthcare utilization*			
Hospital admissions	0.3	0.05	0.503
Emergency department visits	0.62	0.18	0.484
Outpatient face-to-face visits	4.91	6.55	0.2
Primary Care visits	1.15	1.14	0.002
Neurology visits	0.4	1.15	0.581
Psychiatry visits	0.09	0.14	0.053
Geriatrics visits	0.09	0.1	0.01
Cardiology visits	0.41	0.41	0.001
Hospice/palliative care visits	0.03	0	0.025
*Vitals*			
Body Mass Index (kg/m^2^), median value	27.18	26.15	0.192
Diastolic blood pressure (mmHg), median value	72.79	73.35	0.066
Pulse rate (beats/min), median value	73.29	69.8	0.309
Oxygen saturation (SpO_2_, %), median value	96.8	97.36	0.32
Systolic blood pressure (mmHg), median value	132.34	129.98	0.148
Body Mass Index (kg/m^2^), closest-to-index value	27.1	26.09	0.188
Calculated Body Mass Index (kg/m^2^), closest-to-index value	27.07	26.05	0.191
Diastolic blood pressure (mmHg), closest-to-index value	72.81	73.03	0.022
Pulse rate (beats/min), closest-to-index value	73.52	70.05	0.268
Oxygen saturation (SpO_2_, %), closest-to-index value	96.72	97.31	0.278
Systolic blood pressure (mmHg), closest-to-index value	131.32	129.04	0.121
Weight (lbs), closest-to-index value	166.14	165.39	0.019
Respiratory rate (breaths/min), closest-to-index value	17.13	16.79	0.158
Temperature (°F), closest-to-index value	97.79	97.78	0.011
Systolic blood pressure (mmHg), median ≥130, n (%)	352,291 (33.1%)	996 (38.1%)	0.127
Systolic blood pressure (mmHg), median ≥140, n (%)	194,209 (18.2%)	491 (18.8%)	0.142
Systolic blood pressure (mmHg), max ≥160, n (%)	216,238 (20.3%)	408 (15.6%)	0.312
Systolic blood pressure (mmHg), max ≥180, n (%)	90,257 (8.5%)	107 (4.1%)	0.3
Systolic blood pressure (mmHg), min ≤89, n (%)	38,126 (3.6%)	39 (1.5%)	0.207
Oxygen saturation (SpO_2_, %), min ≤91, n (%)	90,064 (8.5%)	71 (2.7%)	0.395
Oxygen saturation (SpO_2_, %), min ≤89, n (%)	40,882 (3.8%)	25 (1.0%)	0.282
Diastolic blood pressure (mmHg), median ≥80, n (%)	147,754 (13.9%)	470 (18.0%)	0.004
Diastolic blood pressure (mmHg), median ≥90, n (%)	23,213 (2.2%)	65 (2.5%)	0.025
Diastolic blood pressure (mmHg), max ≥100, n (%)	81,356 (7.6%)	96 (3.7%)	0.284
Diastolic blood pressure (mmHg), min ≤59, n (%)	216,757 (20.3%)	486 (18.6%)	0.222

Patients prescribed anti-amyloid immunotherapy were generally healthier, with lower prevalence of most comorbidities, including heart failure (3.0% versus 12.9%, SMD 0.371), chronic obstructive pulmonary disease (5.8% versus 14.9%, SMD 0.301), or atrial fibrillation (5.1% versus 14.9%, SMD 0.330). Patients prescribed anti-amyloid immunotherapy also had baseline vital measurements consistent with better overall health status, such as lower blood pressure, lower pulse rates, and lower prevalence of documented hypoxia. In terms of medications, patients prescribed anti-amyloid immunotherapy had lower rates of prescription of many chronic disease medications, notably anticoagulants (4.4% versus 15.1%, SMD 0.368) and antiplatelets (17.6% versus 27.1%, SMD 0.230), insulin (5.4% versus 15.1%, SMD 0.323), atypical antipsychotics (4.1% versus 15.9%, SMD 0.402), and mirtazapine (2.9% versus 7.1%, SMD 0.195). Patients prescribed anti-amyloid immunotherapy had lower rates of preceding emergency department (ED) visits and hospitalizations and higher number of outpatient visits, especially neurology visits. Characteristics of patients receiving only anti-amyloid immunotherapy versus only AChEI/memantine are shown in Supplemental Table 1.

### Characteristics of patients included in safety outcomes sub-cohort

After the application of safety analysis inclusion and exclusion criteria (Figure 1), 401 patients were included in the anti-amyloid immunotherapy group and 274,470 patients were included in the AChEI group. This reduction reflected treatment sequencing and the prespecified exclusions shown in Figure 1 rather than complete-case exclusion; missing covariates were imputed as described above. Patient characteristics before propensity score overlap weighting are shown in Table 2; characteristics after overlap weighting are shown in Supplemental Table 2.

**Table 2.** Characteristics of patients in the safety evaluation sub-cohort before overlap weighting.

Variable	AChEI/memantine (n = 274,470)	Anti-amyloid immunotherapy (n = 401)	SMD
*Demographics*			
Age	75.07	73.51	0.161
Sex			
Female, n (%)	163,307 (59.5%)	223 (55.6%)	0.079
Male, n (%)	111,132 (40.5%)	178 (44.4%)	0.079
*Race*			
American Indian or Alaska Native, n (%)	715 (0.3%)	<11 (<2.7%)	0.072
Asian, n (%)	6757 (2.5%)	<11 (<2.7%)	0.112
Black or African American, n (%)	24,889 (9.1%)	<11 (<2.7%)	0.328
More than one Race, n (%)	26,993 (9.8%)	45 (11.2%)	0.045
Native Hawaiian or Other Pacific Islander, n (%)	269 (0.1%)	<11 (<2.7%)	0.036
Other Race, n (%)	7782 (2.8%)	<11 (<2.7%)	0.158
White, n (%)	200,701 (73.1%)	339 (84.5%)	0.282
Ethnicity			
Hispanic or Latino, n (%)	21,212 (7.7%)	<11 (<2.7%)	0.3
Not Hispanic or Latino, n (%)	253,258 (92.3%)	395 (98.5%)	0.3
Marital status			
Divorced or Legally Separated, n (%)	26,271 (9.6%)	17 (4.2%)	0.211
Married, n (%)	151,296 (55.1%)	309 (77.1%)	0.476
Other, n (%)	10,589 (3.9%)	<11 (<2.7%)	0.111
Unmarried, n (%)	36,587 (13.3%)	31 (7.7%)	0.183
Widowed, n (%)	49,727 (18.1%)	36 (9.0%)	0.269
RUCA group			
Rural, n (%)	13,538 (4.9%)	<11 (<2.7%)	0.178
Suburban, n (%)	27,335 (10.0%)	27 (6.7%)	0.117
Urban, n (%)	233,597 (85.1%)	367 (91.5%)	0.201
U.S. Census division			
East North Central, n (%)	50,079 (18.2%)	52 (13.0%)	0.146
East South Central, n (%)	18,012 (6.6%)	22 (5.5%)	0.045
Middle Atlantic, n (%)	32,884 (12.0%)	63 (15.7%)	0.108
Mountain, n (%)	13,356 (4.9%)	12 (3.0%)	0.097
New England, n (%)	13,987 (5.1%)	31 (7.7%)	0.108
Pacific, n (%)	19,860 (7.2%)	21 (5.2%)	0.083
South Atlantic, n (%)	72,603 (26.5%)	122 (30.4%)	0.088
West North Central, n (%)	16,706 (6.1%)	17 (4.2%)	0.084
West South Central, n (%)	36,983 (13.5%)	61 (15.2%)	0.05
Social Vulnerability Index (percentile, 2020, by ZIP)	0.57	0.45	0.438
*Laboratory variables*			
High-density lipoprotein, mg/dL	57.52	58.59	0.061
Low-density lipoprotein, mg/dL	98.92	97.7	0.033
Serum Triglycerides, mg/dL	119.61	113.18	0.094
Hematocrit (%)	40.88	42.24	0.297
Hemoglobin, mg/dL	13.44	13.95	0.325
White blood cell count, cells/uL	7.12	6.76	0.134
Platelet count, cells/uL	238.51	236.75	0.024
Potassium, mEq/L	4.24	4.29	0.093
Sodium, mEq/L	139.6	139.73	0.046
Serum Creatinine, mg/dL	0.99	0.93	0.147
ALT, u/L	20.15	20.37	0.013
AST, u/L	23.61	22.8	0.046
HbA1c (%)	5.94	5.8	0.152
*Medical history*			
Myocardial infarction, n (%)	7890 (2.9%)	<11 (<2.7%)	0.057
Congestive heart failure, n (%)	14,892 (5.4%)	<11 (<2.7%)	0.182
Peripheral vascular disease, n (%)	21,388 (7.8%)	31 (7.7%)	0.002
Cerebrovascular disease, n (%)	9965 (3.6%)	14 (3.5%)	0.008
Chronic obstructive pulmonary disease (COPD), n (%)	30,178 (11.0%)	23 (5.7%)	0.191
Rheumatologic disease, n (%)	7630 (2.8%)	11 (2.7%)	0.002
Peptic ulcer disease, n (%)	1696 (0.6%)	<11 (<2.7%)	0.112
Mild liver disease, n (%)	8373 (3.1%)	13 (3.2%)	0.011
Diabetes mellitus (no complications), n (%)	47,662 (17.4%)	48 (12.0%)	0.153
Diabetes mellitus with complications, n (%)	25,932 (9.4%)	24 (6.0%)	0.13
Paralysis, n (%)	1335 (0.5%)	<11 (<2.7%)	0.002
Chronic kidney disease, n (%)	32,137 (11.7%)	22 (5.5%)	0.223
Any malignancy (excluding skin), n (%)	25,713 (9.4%)	26 (6.5%)	0.107
Moderate to severe liver disease, n (%)	796 (0.3%)	<11 (<2.7%)	0.076
Metastatic solid tumor, n (%)	5775 (2.1%)	<11 (<2.7%)	0.115
HIV/AIDS, n (%)	380 (0.1%)	<11 (<2.7%)	0.025
Cardiac arrhythmia, n (%)	28,102 (10.2%)	34 (8.5%)	0.06
Depression, n (%)	52,803 (19.2%)	64 (16.0%)	0.086
Drug abuse, n (%)	4938 (1.8%)	<11 (<2.7%)	0.094
Psychoses, n (%)	8255 (3.0%)	18 (4.5%)	0.078
Valvular heart disease n (%)	14,843 (5.4%)	25 (6.2%)	0.035
Alcohol use disorder n (%)	3838 (1.4%)	<11 (<2.7%)	0.013
Alzheimer's Disease diagnosis, n (%)	41,943 (15.3%)	225 (56.1%)	0.942
Anxiety Disorders, n (%)	48,287 (17.6%)	72 (18.0%)	0.009
Chronic pain, n (%)	73,170 (26.7%)	101 (25.2%)	0.034
Epilepsy, n (%)	5867 (2.1%)	<11 (<2.7%)	0.144
Fall-related injury, n (%)	34,678 (12.6%)	47 (11.7%)	0.028
Malnutrition, n (%)	17,276 (6.3%)	15 (3.7%)	0.117
Migraine, n (%)	10,002 (3.6%)	17 (4.2%)	0.031
Osteoarthritis, n (%)	35,600 (13.0%)	48 (12.0%)	0.03
Peripheral artery disease, n (%)	20,802 (7.6%)	24 (6.0%)	0.063
Pneumonia, n (%)	6034 (2.2%)	<11 (<2.7%)	0.148
Urinary Tract Infection, n (%)	16,208 (5.9%)	18 (4.5%)	0.064
*Procedures*			
Coronary artery bypass grafting (CABG), n (%)	63 (0.0%)	<11 (<2.7%)	0.021
Electrical cardioversion, n (%)	20 (0.0%)	<11 (<2.7%)	0.012
Pacemaker, n (%)	1740 (0.6%)	<11 (<2.7%)	0.014
Defibrillator, n (%)	583 (0.2%)	<11 (<2.7%)	0.008
Implantable cardiac monitor, n (%)	319 (0.1%)	<11 (<2.7%)	0.096
Percutaneous coronary intervention (stent), n (%)	855 (0.3%)	<11 (<2.7%)	0.012
Peripheral arterial stent, n (%)	287 (0.1%)	<11 (<2.7%)	0.046
*Prescription drug exposures*			
Angiotensin receptor blocker (ARB), n (%)	54,883 (20.0%)	81 (20.2%)	0.005
ACE inhibitor, n (%)	47,776 (17.4%)	57 (14.2%)	0.088
Calcium channel blocker, n (%)	62,135 (22.6%)	66 (16.5%)	0.156
Beta-blocker, n (%)	76,941 (28.0%)	94 (23.4%)	0.105
Potassium-sparing diuretic, n (%)	10,147 (3.7%)	14 (3.5%)	0.011
Thiazide diuretic, n (%)	36,131 (13.2%)	36 (9.0%)	0.134
Loop diuretic, n (%)	25,951 (9.5%)	16 (4.0%)	0.22
Alpha-1 antagonist, n (%)	4561 (1.7%)	<11 (<2.7%)	0.025
Vasodilator, n (%)	24,795 (9.0%)	28 (7.0%)	0.076
Antiplatelets, n (%)	52,653 (19.2%)	71 (17.7%)	0.038
Statin, n (%)	129,074 (47.0%)	207 (51.6%)	0.092
Fibrates, n (%)	5114 (1.9%)	<11 (<2.7%)	0.027
Bile acid sequestrant, n (%)	3160 (1.2%)	<11 (<2.7%)	0.015
PCSK9 inhibitor, n (%)	1894 (0.7%)	20 (5.0%)	0.261
Ezetimibe, n (%)	10,097 (3.7%)	33 (8.2%)	0.193
Icosapent ethyl, n (%)	1461 (0.5%)	<11 (<2.7%)	0.076
Metformin, n (%)	33,115 (12.1%)	45 (11.2%)	0.026
Insulin, n (%)	29,348 (10.7%)	29 (7.2%)	0.121
GLP-1 Agonists, n (%)	12,596 (4.6%)	20 (5.0%)	0.019
SGLT-2 Inhibitors, n (%)	13,374 (4.9%)	17 (4.2%)	0.03
Sulfonylureas, n (%)	12,316 (4.5%)	<11 (<2.7%)	0.109
DDP-IV Inhibitors, n (%)	7226 (2.6%)	<11 (<2.7%)	0.025
Thiazolidinediones, n (%)	3134 (1.1%)	<11 (<2.7%)	0.031
SSRI, n (%)	69,853 (25.5%)	110 (27.4%)	0.045
SNRI, n (%)	27,070 (9.9%)	46 (11.5%)	0.052
Tricyclic antidepressant (TCA), n (%)	11,050 (4.0%)	<11 (<2.7%)	0.119
Monoamine oxidase inhibitor, n (%)	502 (0.2%)	<11 (<2.7%)	0.061
Buspirone, n (%)	11,592 (4.2%)	22 (5.5%)	0.059
Benzodiazepines, n (%)	68,013 (24.8%)	103 (25.7%)	0.021
Trazodone, n (%)	27,307 (9.9%)	32 (8.0%)	0.069
Bupropion, n (%)	15,582 (5.7%)	32 (8.0%)	0.091
Mirtazapine, n (%)	14,219 (5.2%)	<11 (<2.7%)	0.172
Gaba Analogs, n (%)	48,822 (17.8%)	53 (13.2%)	0.127
Typical Antipsychotics, n (%)	8140 (3.0%)	<11 (<2.7%)	0.045
Atypical antipsychotic, n (%)	31,555 (11.5%)	13 (3.2%)	0.32
Thyroid hormone replacement, n (%)	41,858 (15.3%)	54 (13.5%)	0.051
Bisphosphonate, n (%)	14,252 (5.2%)	20 (5.0%)	0.009
Short-acting β-agonist (SABA), n (%)	47,647 (17.4%)	50 (12.5%)	0.138
Short-acting muscarinic antagonist (SAMA), n (%)	20,869 (7.6%)	17 (4.2%)	0.143
Long-acting β-agonist (LABA), n (%)	18,326 (6.7%)	15 (3.7%)	0.132
Long-acting muscarinic antagonist (LAMA), n (%)	18,121 (6.6%)	15 (3.7%)	0.129
Inhaled corticosteroid, n (%)	38,232 (13.9%)	52 (13.0%)	0.028
Proton pump inhibitor, n (%)	71,832 (26.2%)	92 (22.9%)	0.075
H2 receptor blocker, n (%)	32,068 (11.7%)	52 (13.0%)	0.039
Opioids, n (%)	87,592 (31.9%)	122 (30.4%)	0.032
Penicillin (± beta-lactamase inhibitor), n (%)	32,050 (11.7%)	56 (14.0%)	0.068
First Generation Cephalosporins, n (%)	40,233 (14.7%)	47 (11.7%)	0.087
2nd-generation cephalosporin, n (%)	5023 (1.8%)	<11 (<2.7%)	0.012
3rd-generation cephalosporin, n (%)	16,031 (5.8%)	20 (5.0%)	0.038
Fourth Generation Cephalosporins, n (%)	1449 (0.5%)	<11 (<2.7%)	0.045
Fifth Generation Cephalosporins, n (%)	<11 (<2.7%)	<11 (<2.7%)	0.005
Carbapenem, n (%)	535 (0.2%)	<11 (<2.7%)	0.012
Monobactam, n (%)	68 (0.0%)	<11 (<2.7%)	0.022
Macrolide, n (%)	22,925 (8.4%)	42 (10.5%)	0.073
Fluoroquinolones, n (%)	28,317 (10.3%)	40 (10.0%)	0.011
Tetracycline, n (%)	23,623 (8.6%)	43 (10.7%)	0.072
Aminoglycoside, n (%)	4340 (1.6%)	<11 (<2.7%)	0.048
Sulfonamide, n (%)	1466 (0.5%)	<11 (<2.7%)	0.005
Lincosamide, n (%)	7637 (2.8%)	16 (4.0%)	0.067
Glycopeptide, n (%)	8721 (3.2%)	<11 (<2.7%)	0.132
Oxazolidinone, n (%)	750 (0.3%)	<11 (<2.7%)	0.036
Nitroimidazole, n (%)	9334 (3.4%)	16 (4.0%)	0.031
*Healthcare utilization*			
Hospital admissions, mean	0.12	0.03	0.259
ED Visits, mean	0.34	0.18	0.214
Outpatient face-to-face visits, mean	4.96	8.03	0.375
Primary care visits, mean	1.16	1.18	0.012
Neurology visits, mean	0.5	1.1	0.435
Psychiatry visits, mean	0.12	0.12	0.002
Geriatrics visits, mean	0.06	0.08	0.017
Cardiology visits, mean	0.27	0.57	0.176
*Vitals*			
Body Mass Index (kg/m^2^), median value	26.85	26.5	0.063
Diastolic blood pressure (mmHg), median value	72.89	73.9	0.122
Pulse rate (beats/min), median value	72.45	70.98	0.128
Oxygen saturation (SpO_2_, %), median value	96.51	97.08	0.232
Systolic blood pressure (mmHg), median value	133.47	129.88	0.235
Body Mass Index (kg/m^2^), closest-to-index value	26.79	26.48	0.055
Diastolic blood pressure (mmHg), closest-to-index value	72.78	73.41	0.062
Pulse rate (beats/min), closest-to-index value	72.57	71.42	0.088
Oxygen saturation (SpO_2_, %), closest-to-index value	96.46	97.01	0.213
Systolic blood pressure (mmHg), closest-to-index value	132.75	129.07	0.199
Systolic blood pressure (mmHg), median ≥130, n (%)	147,265 (53.7%)	184 (45.9%)	0.156
Systolic blood pressure (mmHg), median ≥140, n (%)	84,782 (30.9%)	83 (20.7%)	0.235
Systolic blood pressure (mmHg), max ≥160, n (%)	85,894 (31.3%)	75 (18.7%)	0.294
Systolic blood pressure (mmHg), max ≥180, n (%)	24,415 (8.9%)	21 (5.2%)	0.143
Systolic blood pressure (mmHg), min ≤89, n (%)	6773 (2.5%)	<11 (<2.7%)	0.137
Oxygen saturation (SpO_2_, %), min ≤91, n (%)	50,846 (18.5%)	30 (7.5%)	0.333
Oxygen saturation (SpO_2_, %), min ≤89, n (%)	7542 (2.7%)	<11 (<2.7%)	0.179
Diastolic blood pressure (mmHg), median ≥80, n (%)	52,925 (19.3%)	102 (25.4%)	0.148
Diastolic blood pressure (mmHg), median ≥90, n (%)	6951 (2.5%)	<11 (<2.7%)	0.002
Diastolic blood pressure (mmHg), max ≥100, n (%)	15,984 (5.8%)	12 (3.0%)	0.138
Diastolic blood pressure (mmHg), min ≤59, n (%)	72,613 (26.5%)	84 (20.9%)	0.13

### Primary end point

Crude and adjusted rates of ICH are shown in Figure 2. Crude and adjusted survival curves are shown in Supplemental Figure 1. The crude rate of intracranial hemorrhage was 3.82 per 1000 person-years in the AChEI/memantine group and 2.95 in the anti-amyloid immunotherapy group (hazards ratio (HR) 0.77, 95% CI 0.11–5.46). The overlap-weighting adjusted rate of intracranial hemorrhage was 4.00 per 1000 person-years in the AChEI/memantine group and 2.97 in the anti-amyloid immunotherapy group (overlap-weighted HR (owHR) 0.73, 95% confidence interval (CI) 0.10–5.12). The AChEI/memantine group had 933 ICH events; the anti-amyloid group had fewer than 11 events. Crude person-time is reported in Figure 2.

**Figure 2.**
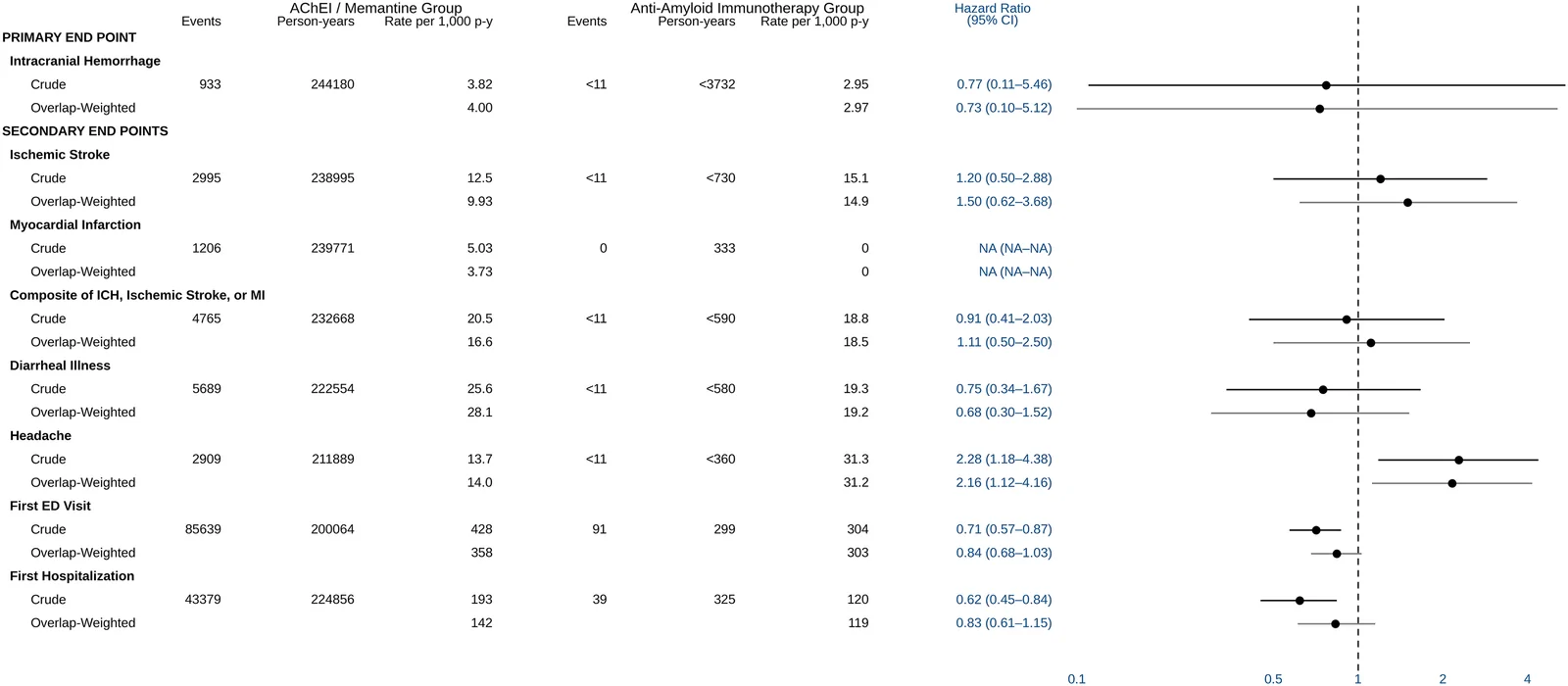
Crude and adjusted rates and hazard ratios for the primary and secondary study end points.

### Secondary end points

There was no association between anti-amyloid immunotherapy prescription and rates of ischemic stroke, myocardial infarction, or the composite of ICH, ischemic stroke, or myocardial infarction (MI) before or after overlap weighting (Figure 2). Crude and adjusted survival curves are shown in Supplemental Figure 1. Furthermore, there was no association between anti-amyloid immunotherapy and diagnosis of diarrheal illness before or after overlap weighting (Figure 2). There was an increased rate of headache (owHR 2.28, ([95% CI 1.18–4.38]; adjusted rate 14.0 per 1000 p-y versus 31.2 per 1000 p-y, adjusted rate difference 17.2 per 1000 p-y, number needed to harm (NNH) of 58) after overlap weighting (Figure 2). Anti-amyloid immunotherapy prescription was associated with lower hazard of emergency department visit (HR 0.71, 95% CI 0.57–0.87) and hospitalization (HR 0.62, 95% CI 0.45–0.84) before adjustment, but after adjustment these differences were attenuated (owHR 0.84, 95% CI 0.68–1.03 and owHR 0.83, 95% CI 0.61–1.15 respectively).

Patterns of healthcare utilization are shown in Supplemental Table 3. Anti-amyloid immunotherapy prescription was associated with lower unadjusted rates of ED visits (RR 0.71, 95% CI 0.57–0.87) and hospitalizations (RR 0.62, 95% CI 0.45–0.84) However there was no association between anti-amyloid immunotherapy prescription and either ED visits (owRR 0.83, 95% CI 0.68–1.03) or hospitalizations (owRR 0.83, 95% CI 0.61–1.15) after overlap weighting.

### Exploratory end points

There was no association between anti-amyloid immunotherapy prescription and myocarditis/pericarditis, severe skin reactions, thyroid dysfunction, hyperosmolar hyperglycemic state/diabetic ketoacidosis, or acute liver injury, although estimates were imprecise (Table 3). After overlap weighting, there was a higher rate of respiratory tract infections among patients prescribed anti-amyloid immunotherapy (owHR 1.57 [95% CI 1.04–2.37]; adjusted rate 85.1 1000 p-y versus 53.6 per 1000 p-y, adjusted rate difference 31.5 per 1000 p-y, NNH 32) (Figure 3).

**Table 3.** Exploratory and falsification endpoints for the study.

	AChEI/memantine			Anti-amyloid immunotherapy			
Outcome	Number of events	Number of person-years	Event rate	Number of events	Number of person-years	Event rate	Hazard Ratio (95% CI)
Respiratory Infection							
Crude	11,060	204,563	54.07	23	269	85.60	1.58 (1.05–2.37)
Overlap-weighted			53.64			85.05	1.57 (1.04–2.37)
Thyroid disorders							
Crude	1798	201,669	8.92	<11	<1537	7.16	0.80 (0.20–3.20)
Overlap-weighted			7.81			7.12	0.90 (0.22–3.59)
Interstitial lung disease							
Crude	60	245,129	0.24	0	<280	0	NA
Overlap-weighted			0.14			0	NA
Severe skin disorders							
Crude	252	241,880	1.04	0	<280	0	NA
Overlap-weighted			1.05			0	NA
Acute liver injury							
Crude	108	244,722	0.44	<11	<280	0	NA
Overlap-weighted			0.23			0	NA
Hyperosmolar Hyperglycemic State or DKA							
Crude	395	243,148	1.62	0	<280	0	NA
Overlap-weighted			0			0	NA
Myocarditis or Pericarditis							
Crude	23	245,360	0.09	0	<280	0	NA
Overlap-weighted			0.11			0	NA
Falsification Endpoint							
Cataract							
Crude	1838	229,664	8.00	<11	3506	3.14	0.39 (0.05–2.77)
Overlap-weighted			7.94			3.04	0.42 (0.06–2.94)

**Figure 3.**
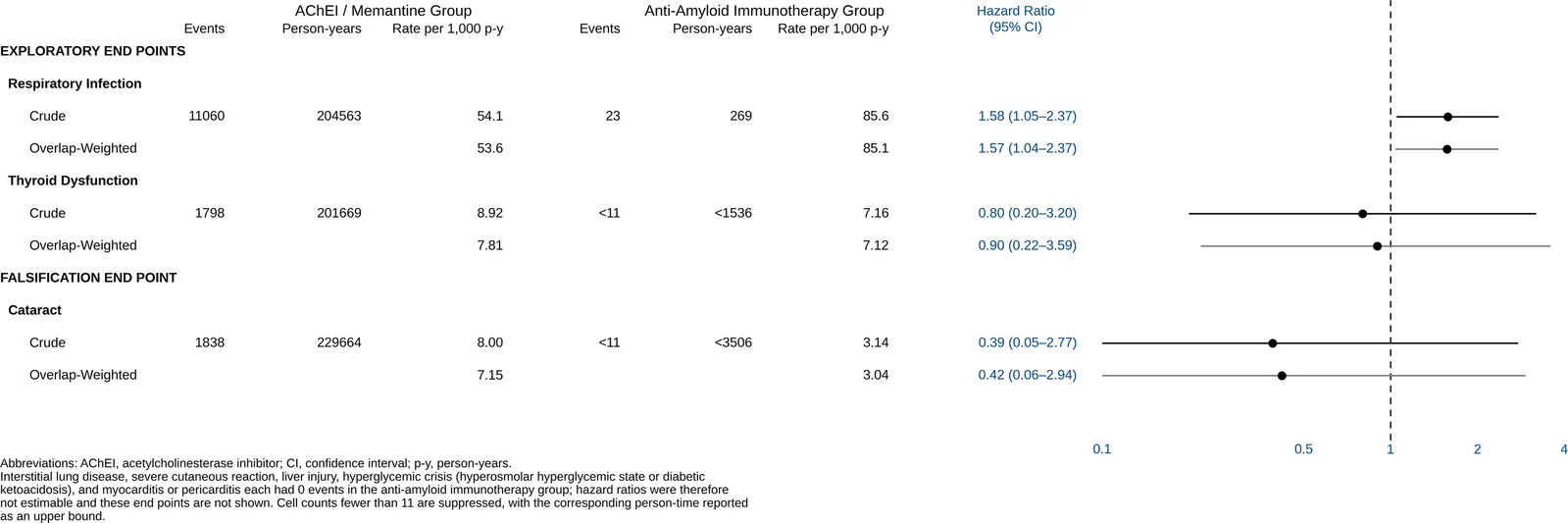
Crude and adjusted associations for the exploratory and falsification endpoints.

### Quantitative bias analysis/sensitivity analyses

The E-value for the point estimate of the primary end point to reveal a true hazard ratio of 2.0 was 4.92, suggesting that results were robust to unmeasured confounding.^25^ The E-value for the point estimate for the headache endpoint was 3.99, and the E-value for the respiratory tract infection point estimate was 2.52. The falsification end point of cataract revealed a similar rate of cataract among patients in the two groups before and after overlap weighting (Figure 3). Sensitivity analyses using a clone-censor-weight approach to account for treatment switching are shown in Supplemental Table 4, and results were consistent with the primary analysis, although the associations with headache and respiratory tract infections were not observed in this analysis.^17^ A sensitivity analysis restricting to patients with age <75 is shown in Supplemental Table 5 and was similar to the primary analysis.

## Discussion

In this nationwide cohort study of 2616 patients who received anti-amyloid immunotherapy from July 1, 2023 to January 1, 2025, we observed significant demographic differences in patient adoption. Compared with patients receiving standard oral dementia medications (AChEI/memantine alone), those prescribed anti-amyloid immunotherapy were healthier at baseline and more likely to be male, white, and from socioeconomically advantaged areas. We did not detect a statistically significant association between anti-amyloid immunotherapy and intracranial hemorrhage, ischemic stroke, or myocardial infarction. However, the small, treated safety cohort and low event counts produced wide confidence intervals, and the results should be interpreted as an inability to detect a statistically significant increase rather than evidence definitively confirming safety. The ICH endpoint addresses clinically coded hemorrhage and does not characterize the broader MRI-defined ARIA spectrum. The safety analysis did, however, find a two-fold increased risk of headaches (a known adverse effect of anti-amyloid immunotherapy) and a modestly increased risk of respiratory infection, both of which merit further study.

Our findings highlight an important disparity in the real-world use of anti-amyloid immunotherapy in the United States. Prior work indicates that eligibility criteria based on amyloid biomarkers may disproportionately exclude certain racial and ethnic groups, such as Hispanic and Black patients.^26^ This is further supported by our finding of an underrepresentation of female, Hispanic, and Black patients in the immunotherapy cohort, and is consistent with findings from the AHEAD preclinical Alzheimer's disease program demonstrating lower plasma amyloid eligibility in underrepresented racial and ethnic groups.^27^ Some evidence suggests that Aβ pathology and treatment effect of anti-amyloid immunotherapy may vary by sex, and that female patients may have earlier tau deposition than male patients.^28–30^ Additionally, access to medical care, acceptability of therapy, timeliness of diagnosis, or other social and environmental factors could impact therapy adoption.^11,14,31–35^ Given the low observed prescription of anti-amyloid immunotherapy among minoritized patient populations, this study is limited in its ability to evaluate distinct safety signals in these groups. The observed selection pattern also limits generalizability: safety estimates from this relatively healthy, urban, and socioeconomically advantaged cohort may not apply to older or frailer patients, rural populations, socioeconomically disadvantaged patients, underrepresented racial and ethnic groups, or patients with comorbidity profiles that complicate treatment and MRI monitoring.

The observed safety results did not identify statistically significant increases in intracranial hemorrhage or ischemic stroke, but the estimates were imprecise and should not be interpreted as definitively establishing safety. The study was unable to directly evaluate ARIA-H or ARIA-E. Because intracranial hemorrhage is a coarse, code-based surrogate, isolated ARIA-H microhemorrhages or superficial siderosis, ARIA-E without hemorrhage, and imaging abnormalities without a coded clinical diagnosis would be missed. This outcome misclassification may bias the association toward the null and prevents estimation of overall ARIA incidence or exclusion of clinically important imaging-detected toxicity. Clinical trials indicate that many ARIA events are asymptomatic or cause only minor symptoms, underscoring why a coded-ICH endpoint cannot capture the full treatment-related safety burden.^36–38^ This study excluded a number of patients at high risk for ARIA based on established factors, such as antithrombotic use.^37,39^ Therefore, our findings should be interpreted with caution, as many patients with clear indications for anticoagulation or a history of anticoagulant therapy were excluded from the immunotherapy group. The higher headache rate is consistent with pivotal trials, current product labeling, and post-marketing pharmacovigilance reports. Because headache may reflect an infusion reaction, ARIA, or a nonspecific symptom, new or changing headache during treatment should prompt clinical assessment and MRI when ARIA is suspected.^36,39^

Our study did not find an increased risk of diarrheal illness, which is a known adverse effect of anti-amyloid immunotherapy. This finding is likely due to our use of AChEIs, which are classically associated with diarrhea, as the active control.^40^ Thus, our study did not detect an increased risk of diarrheal illness with anti-amyloid immunotherapy compared with a common alternative treatment. Additionally, we investigated a broad range of immune-related safety signals. Aside from respiratory tract infections, we found no new safety signals associated with anti-amyloid immunotherapy. The higher respiratory-infection rate observed in the primary overlap-weighted analysis is compatible with events reported in pivotal trials, although infection definitions and the COVID-19-era timing of some trial follow-up complicate direct comparison.^2,4^ Available post-marketing safety studies have focused primarily on ARIA, infusion-related reactions, and other neurologic events; spontaneous reports cannot estimate incidence or establish causality. Given that immunomodulatory therapies are consistently associated with elevated risk of respiratory symptoms,^41^ the respiratory-infection signal should be considered hypothesis-generating. The association was not reproduced in the clone-censor-weight sensitivity analysis and may reflect residual confounding, differential ascertainment, chance, or a treatment effect; confirmation in larger cohorts with longer follow-up is needed.

This study has several limitations. First, identifying a suitable active comparator for anti-amyloid immunotherapy is challenging. AChEI/memantine are used in a wider range of patient populations, including patients without Alzheimer's disease and those with more advanced cognitive impairment than patients eligible for anti-amyloid immunotherapy. Since cognitive measures are generally unavailable in semi-structured electronic health records, our study could not control for cognitive stage at entry and could accordingly not estimate the comparative effectiveness of anti-amyloid immunotherapy. *APOE* ε4 status, baseline MRI findings (including cerebral microhemorrhages and cerebral amyloid angiopathy), baseline cognitive severity, and functional status were not reliably available in electronic health record data. Because these variables can influence both treatment selection and ARIA or hemorrhage risk, confounding by eligibility, indication, or contraindication may persist after overlap weighting. Future studies using natural language processing tools to extract cognitive testing results from unstructured electronic health records could help extend the impact and rigor of observational comparative research into these questions. Second, concurrent therapy use was very common, with most patients receiving anti-amyloid immunotherapy also receiving AChEI/memantine. This was generally prior to the initiation of anti-amyloid immunotherapy, and results were similar with a clone-censor-weight approach. Third, our study could not directly evaluate ARIA-H or ARIA-E, adverse effects unique to anti-amyloid immunotherapy that are monitored by MRI in routine clinical practice. Because standardized longitudinal neuroimaging was unavailable, we also could not assess MRI-defined edema, serial brain-volume change, or imaging-based atrophy after treatment. Future prospective studies should include standardized baseline and follow-up MRI with volumetric measures to characterize edema, ARIA, and longitudinal brain-volume change and to distinguish treatment-related volume change from neurodegenerative atrophy. Prospective studies incorporating baseline and follow-up FDG-PET could additionally evaluate changes in cerebral metabolism, treatment efficacy, and potential functional injury. Fourth, our study only included patients seen at health systems using the Epic electronic health record system. This may limit generalizability to other clinical settings. Events occurring outside of Epic Health systems were also not available in our dataset. Fifth, despite the largest nationwide sample to date, this study was underpowered to detect small but clinically meaningful safety signals. This is due to low event rates in the anti-amyloid immunotherapy group and short, variable follow-up given the recent approval of these therapies; therefore, the study was unable to detect or exclude clinically important increased risk.

In conclusion, this study found significant demographic differences in the early adoption of anti-amyloid immunotherapy. The study did not detect a statistically significant increase in intracranial hemorrhage or ischemic stroke, but low event counts and imprecise estimates preclude confirmation of safety and do not exclude clinically important harm. For clinicians, these results support continued adherence to product-specific eligibility criteria, individualized ARIA risk assessment, baseline and scheduled MRI monitoring, and prompt evaluation of new neurologic symptoms; they should not be used to relax established safety precautions. The observed increased risk of headache and respiratory-infection warrant clinical vigilance and further study.

## Supplemental Material

sj-pdf-1-alr-10.1177_25424823261483526 - Supplemental material for Uptake and safety profile of anti-amyloid immunotherapies in routine clinical practice Supplemental material, sj-pdf-1-alr-10.1177_25424823261483526 for Uptake and safety profile of anti-amyloid immunotherapies in routine clinical practice by Jay B. Lusk, Kate Vinita Fitch, Kim G. Johnson, Andy Liu, Jennifer L. Lund, Laine E. Thomas, Ryan McDevitt, Anqi Zhao, Heather E. Whitson, Richard O'Brien, Shannon Aymes, Bradley G. Hammill, Brian Mac Grory, Fan Li and Emily C. O'Brien in Journal of Alzheimer's Disease Reports

sj-docx-2-alr-10.1177_25424823261483526 - Supplemental material for Uptake and safety profile of anti-amyloid immunotherapies in routine clinical practice Supplemental material, sj-docx-2-alr-10.1177_25424823261483526 for Uptake and safety profile of anti-amyloid immunotherapies in routine clinical practice by Jay B. Lusk, Kate Vinita Fitch, Kim G. Johnson, Andy Liu, Jennifer L. Lund, Laine E. Thomas, Ryan McDevitt, Anqi Zhao, Heather E. Whitson, Richard O'Brien, Shannon Aymes, Bradley G. Hammill, Brian Mac Grory, Fan Li and Emily C. O'Brien in Journal of Alzheimer's Disease Reports

sj-pdf-3-alr-10.1177_25424823261483526 - Supplemental material for Uptake and safety profile of anti-amyloid immunotherapies in routine clinical practice Supplemental material, sj-pdf-3-alr-10.1177_25424823261483526 for Uptake and safety profile of anti-amyloid immunotherapies in routine clinical practice by Jay B. Lusk, Kate Vinita Fitch, Kim G. Johnson, Andy Liu, Jennifer L. Lund, Laine E. Thomas, Ryan McDevitt, Anqi Zhao, Heather E. Whitson, Richard O'Brien, Shannon Aymes, Bradley G. Hammill, Brian Mac Grory, Fan Li and Emily C. O'Brien in Journal of Alzheimer's Disease Reports

## Data Availability

Data can be obtained from Epic Systems under an approved institutional Data Use Agreement for Epic Cosmos.
