# Supplementary material for: Uptake and safety profile of anti-amyloid immunotherapies in routine clinical practice
Source: J Alzheimers Dis Rep. 2026 Sep 30;10:25424823261483526. doi: 10.1177/25424823261483526 (PMC13662371; doi:10.1177/25424823261483526)
Supplement: Supplementary file 1 [file sj-pdf-1-alr-10.1177_25424823261483526.pdf]

Anti-Amyloid Therapy Safety Evaluation SAP

Objective: To compare the frequency of adverse events after treatment initiation with anti-amyloid monoclonal antibody therapy versus approved dementia medications (donepezil, memantine, rivastigmine, or galantamine).

Population: Patients with dementia potentially eligible for dementia pharmacotherapy. The inclusion criterion will be first prescription of either lecanemab or donanemab (Arm 1) or donepezil, memantine, rivastigmine, or galantamine (Arm 2). The study will use a new-user active comparator design using target trial emulation principles. Accordingly, patients will be classified according to the group they are prescribed first to concord with the intent to treat principle. Patients with first prescription between 07/01/2023 and 01/01/2025 will be included.

Exclusion Criteria: 1) History of ischemic or hemorrhagic stroke; 2) History of atrial fibrillation; 3) Cardiac valvular surgery in lookback period; 4) Prescription for warfarin or any non-vitamin K oral anticoagulant in the lookback period; 5) Deep vein thrombosis. 6) Encounter for hospice/palliative care services in lookback period; 7) Age at index >=90 years. Codes used to identify exclusion criteria are shown in **APPENDIX 1.**

End Points: The primary end point will be time to first diagnosis of nontraumatic intracerebral hemorrhage, defined as I61.* or I62.* This endpoint was selected due to the known risk of amyloid-related imaging abnormalities (ARIA), which does not have a specific ICD-10 code, and is most likely to be captured as intracranial hemorrhage in electronic health record data. Codelists for each primary, secondary, and exploratory endpoint are shown in **APPENDIX 2.** For all endpoints, we will require the diagnosis code of interest in the primary diagnosis position and will not use problem list derived diagnoses. Participants with a history of each endpoint (except healthcare utilization endpoints) in the lookback window will be excluded, to avoid potential misclassification of delayed management of an incident event as a subsequent recurrent event.

Secondary endpoints will include the following.

1) Cardiovascular endpoints: myocardial infarction, ischemic stroke, and composite of myocardial infarction, ischemic stroke, and intracranial hemorrhage
2) Diagnosis of diarrheal illness
3) Diagnosis of headache
4) Healthcare utilization endpoints: hospitalization and emergency department visit, modeled both as a time-to-event endpoint and as a count endpoint with poisson or negative binomial regression with a 1-year offset depending on the degree of overdispersion.

The above secondary endpoints were selected as they are either 1) major preventable causes of death among the general population with dementia that could be associated with amyloid immunotherapy (i.e. cardiovascular safety endpoints); 2) Established harms of amyloid immunotherapy observed in randomized trials; 3) General healthcare utilization endpoints that reflect broader surrogate of potential harms that could manifest with a need for increased acute healthcare utilization.

Exploratory safety endpoints, based on adverse effects of other immunotherapies but not seen in existing clinical trials of amyloid immunotherapies will include the following as time-to-event endpoints

1) Lower or upper respiratory tract infection
2) Myocarditis or pericarditis
3) Acute liver injury
4) Interstitial lung disease
5) Severe skin reactions (lichen dermatitis, psoriasis, bullous pemphigoid, or SJS-TEN)
6) Hypo- or hyperthyroidism
7) Hyperosmolar hyperglycemic state or diabetic ketoacidosis

Adjustment Covariates:

We will use a broad range of adjustment covariates to address measured confounding, using a two-year lookback period. Specific adjustment covariates include:

1) Demographics. Age, Sex, Race, Ethnicity.
2) Social Determinants of Health. SVI percentile, ADI
3) Physical measurements. Systolic blood pressure, diastolic blood pressure, body mass index, height, weight, heart rate, oxygen saturation, and pulse. Physical measurements will be summarized as closest to index date, median in lookback, min in lookback, max in lookback, and range in lookback, to account for biologically meaningful variation in these frequently measured variables.
4) Laboratory measurements. Hemoglobin, hematocrit, white blood cell count, platelet count, aspartate aminotransferase, alanine aminotransferase, serum creatinine, sodium, potassium, glycated hemoglobin, low density lipoprotein cholesterol, high density lipoprotein cholesterol, serum triglycerides. Laboratory measurements will be selected as closest to index date within the 2-year lookback period.
5) Procedures. Coronary artery bypass grafting, carotid endarterectomy, cardiac ablation, pacemaker placement, percutaneous coronary intervention (codelist shown in **APPENDIX 3)**

6) Diagnoses. The individual components of the Elixhauser comorbidity index and claims-based frailty index, as well as history of alcohol use disorder, anxiety disorders, chronic pain, fall-related injury, epilepsy, malnutrition/unintended weight loss, migraine headache, osteoarthritis, peripheral arterial disease, pneumonia, and urinary tract infection (codelists shown in **APPENDIX 4).**
7) Common medications which either reflect general medical illness, are associated with bleeding, or are associated with cardiovascular disease (see **APPENDIX 5**).
8) Healthcare utilization. Number of outpatient encounters, emergency department encounters, hospitalizations as well as number of visits to neurologists, geriatricians, primary care physicians, and cardiologists.

Implausible laboratory measurements will be systematically excluded according to the approach detailed in **APPENDIX 6.**

Treatment of Missing Data: Missing data will be imputed using random forest-based imputation approach[1] using the MissRanger package, as this approach has been shown to be particularly well-suited to very high dimensional data of varying data types. We will interrogate missingness of data elements prior to performing imputation and will exclude variables with extremely high missing rates or those that are nearly universally present or absent (>99%). We will use a maximum number of tress of 100 and three candidate non-missing values to sample for predictive mean matching.

Propensity Score Methods: We will use propensity score overlap weighting.[1,2] If variables have near zero variance (defined systematically with the nearZeroVar function in the *caret* package in R) they will be excluded from propensity score derivation and this will be noted in a supplement.

Statistical Analysis. Cox proportional hazard models will be used for time to event endpoints. The maximum duration of follow-up will be set to one year. Patients will be censored at death, end of data availability, or one year after index date. Cause-specific hazards will be used, as Epic Cosmos death data is not complete making methods used to treat death as a competing risk difficult to reliably implemented.

Quantitative Bias Analysis.

1) E-values. E-values will be calculated for the primary and secondary endpoints.
2) Falsification endpoint. Time to diagnosis of cataract will be used as a negative control (falsification) endpoint, as cataract is a common age-related condition which requires health system engagement to address and accordingly shares underlying confounding factors with both exposures, but is not biologically linked to

either exposure. The codes used for cataract are adapted from Muir et al.[3] after conversion to ICD-10-CM, corresponding to H25.*, H26.*, H27.*, and H28.*.

3) Sensitivity analysis restricting to age <75 in both arms, to assess unmeasured confounding by disease severity which is most closely related to age
4) Sensitivity analysis using clone-censor-weighting (if feasible given number of patients in each potential treatment strategy) to emulate
    a. Strategy of addition of amyloid therapy on baseline acetylcholinesterase inhibitor therapy. In this design, the index date is the date of first prescription of acetylcholinesterase therapy. Patients never receiving acetylcholinesterase inhibitors will be excluded.
    b. Strategy of addition of acetylcholinesterase inhibitor therapy on baseline anti-amyloid therapy. In this design, the index date is the date of first prescription of anti-amyloid therapy. Patients never receiving anti-amyloid therapy will be excluded.
    c. In both designs above, patients will be censored at the time of secondary therapy initiation in one cloned arm, and inverse probability of censoring weighting will be used to fit a weighted cox model, where the final weights are the inverse probability of censoring weights multiplied by the overlap weights.

APPENDIX 1. Codes used to identify study exclusion criteria

Criterion	Codelist
History of ischemic stroke or ICH	Ischemic Stroke (Birman-Deych), G45.0, G45.1, G45.2, G45.8, G45.9, G46.0, G46.1, G46.2, I63.*, I67.81, I67.82, I67.84, I67.89, I67.9*, I69.*; ICH (Birman-Deych): @06.*, I60.*-I62.*
History of atrial fibrillation or atrial flutter	I48.0*-I48.2*, I48.91 or I48.3*, I48.4, I48.92
Cardiac valvular surgery	CPT 33361:33478
Prescription for warfarin or non-vitamin K oral anticoagulant	Generic name %like% “warfarin” or “apixaban” or “rivaroxaban” or “dabigatran” or “edoxaban”
Deep Vein Thrombosis	I80.1*-I80.3*, I80.9*, I82.1*, I82.210, I82.22, I82.290, I82.6*, I82.890, I82.9*, I82.A1, I82.B1, I82.C1 (Birman-Deych)
Hospice/Palliative Care Encounter	Department specialty of Palliative Medicine, Inpatient Hospice, Hospice Services, Home Hospice, or Hospice and Palliative Medicine

APPENDIX 2. Codes used to identify the primary, secondary, and exploratory endpoints.

Endpoint	Codelist
Intracerebral hemorrhage	I61.* or I62.*
Myocardial infarction	I21.* and I22.* (Metcalfe et al.[4])
Ischemic stroke	I63.* (Hsieh et al.[5])
Diarrheal Illness	R19.7, A09.*, K52.9*,
Headache	G43.*, G44.*, R51.*
Respiratory tract infection	J10-J18 (inclusive), Skull et al.[6]; J00-J06
Myocarditis or pericarditis	A381, A3952, B2682, B3320, B3322, B3324, B5881, D8685, I012, I090, I400, I401, I408, I409, I41, I514, J1082 or J1182 (Wu et al.[7])
Acute liver injury	K71.0, K71.1, K71.2, K71.6, K71.9, K72.0, K72.9, K75.9, K76.2 (Timmer et al.)[8]
Interstitial Lung Disease	J84.112 (Herberts et al.)[9]
Severe skin reactions	L51.1-L51.3 (Wasuwanich et al.[10]), L12.0 (Leisti et al.) [10], L40.0-40.9, M07.0, M07.1, M07.2, M07.3, M09.0 (Ham et al.[11]), L66.1, L43.0, L43.1, L43.2, L43.3, L43.8, L43.9 (del Toro et al)[12]
Hypo- or hyperthyroidism	E01.0, E01.1, E01.2, E01.8, E02.9, E03.2, E03.3, E03.8, E03.9, E06.0, E06.1, E06.2, E06.3, E06.4, E06.5, E06.9, E89.0, E89.0A, E89.0B, E89.0X (Bengtsson et al.[13]), E05.0, E05.1, E05.2, E05.8, E05.9 (Peng et al.)[14]
Hyperosmolar hyperglycemic state or diabetic ketoacidosis	E10.10, E11.10, E13.10, and E14.10 (DKA, Hodzic-Santor et al.[15]), E11.01, E11.00, E13.00, E08.00, E11.10

APPENDIX 3: Codes used to identify procedural adjustment variables.

Procedure	Codelist (CPT unless otherwise specified)
Coronary artery bypass grafting	33510, 33511, 33512, 33513, 33514, 33516, 33517, 33518, 33519, 33521, 33522, 33523, 33530, 33533, 33534, 33535, 33536
Carotid endarterectomy	33501
Pacemaker	33206, 33207, 33208, 33227, 33228, 33214, 33212, 33213, 33,229 93280, 93279, 93288, 93281
Implantable defibrillator	33249, 33240, 33230, 33231, 33262, 33263, 33264, 33270, 93289, 93282, 93283, 93284, 93641
Implantable cardiac monitor	33282, 33284, 93285, 93291, 93298
Percutaneous coronary intervention	92928, 92929, 92933, 92934, 92937, 92941, 92943, 92944; HCPCS C9600, C9601, C9602, C9603, C9604, C9605, C9606, C9607, C9608
Peripheral artery stenting	CPT 37221-37223; 37226-37227; 37230-37235; 37236-37237; 37238-37239; HCPCS C1874-C1877, C2617, C2625

APPENDIX 4: Codes used to identify diagnosis-based adjustment variables.

Condition	Codelist (ICD-10-CM)
Alcohol use disorder	F10.10, F10.11, F10.120, F10.129, F10.20, F10.21, F10.220, F10.229
Anxiety disorders	F06.4*, F40.00, F40.01, F40.02, F40.10, F40.11, F40.210, F40.218, F40.220, F40.228, F40.230, F40.231, F40.232, F40.233, F40.240-F40.243, F40.248, F40.290, F40.291, F40.298, F40.8*, F40.9*, F41.0*, F41.1*, F41.3*, F41.8*, F41.9*, F42.*, F43.0*, F43.10, F43.11, F43.12, F44.9*, F45.8*, F48.8*, F48.9*, F93.8*, F99.*, R45.2*, R45.5*, R45.6*, R45.7*
Chronic Pain	F45.41, F45.42, , G89.0*, G89.21, G89.22, G89.28, G89.29, G89.4*, M25.50, M25.51, M25.55-M25.57, M25.78, M43.2*, M43.6*, M43.8*9, M45.9, M46.1*, M46.41-M46.47, M46.9*, M47.0*, M47.02, M47.1*, M47.24-M47.28, M47.814, M47.815, M47.816, M47.817, M47.818, M47.819, M47.894-M47.898, M48.00, M48.04-M48.08, M48.1*, M48.9*, M50.1*, M50.20, M50.3*, M50.8*, M50.9*, M51.04-M51.06, M51.1*, M51.24, M51.25, M51.2*, M51.4*, M51.8*, M51.9*, M53.1*, M53.3, M53.2*7, M53.2*8, M53.3*, M53.8*, M53.9*, M54.*, M60.8*, M60.9*, M62.830, M67.88, M79.0, M72.9*, M79.1*, M79.2*, M79.6*, M79.7*, M96.1*, M99.22-M99.29, M99.32-M99.39, M99.42-M99.49, M99.52-M99.59, M99.62-M99.69, M99.72-M99.79
Epilepsy	G40.*
Fall-related injury	M48.4*, M48.5*, M67.90, M80.*, M84.*, M99.1*, S00.*-S99.*, T07.XXXS, T14.8XXA, T14.8XXS, T14.90XA, T14.90XS, T14.91XS, T15.00XS, T15.01XS, T15.02XS, T15.10XS, T15.11XS, T15.12XS, T15.80XS, T15.81XS,T15.82XS, T15.90XS, T15.90XS, T15.91XS, T15.92XS, T16.1XXS, T16.2XXS, T16.9XXS,T16.9XXS, T17.0XXS, T17.1XXS, T17.1XXS, T17.200S, T17.208S, T17.210S, T17.218S, T17.220S, T17.228S, T17.290S, T17.298S, T17.300S, T17.308S, T17.310S, T17.318S, T17.320S, T17.328S, T17.390S, T17.398S, T17.400S, T17.408S, T17.410S, T17.418S, T17.420S, T17.428S, T17.490S,

	T17.498S, T17.500S, T17.508S, T17.510S, T17.518S, T17.520S, T17.528S, T17.590S, T17.598S, T17.800S, T17.808S, T17.810S, T17.818S, T17.820S, T17.828S, T17.890S, T17.898S, T17.900S, T17.900S, T17.908S, T17.910S, T17.918S, T17.920S, T17.928S, T17.990S, T17.998S, T18.0XXS, T18.0XXS, T18.100S, T18.108S, T18.110S, T18.118S, T18.120S, T18.128S, T18.190S, T18.198S, T18.2XXS, T18.3XXS, T18.4XXS, T18.5XXS, T18.8XXS, T18.9XXS, T18.9XXS, T19.0XXS, T19.1XXS, T19.2XXS, T19.3XXS, T19.4XXS, T19.8XXS, T19.9XXS, T19.9XXS, T79.0XXS, T79.1XXS, T79.2XXS, T79.4XXS, T79.5XXS, T79.6XXS, T79.7XXS, T79.8XXS, T79.9XXS, T79.9XXS, T79.A0XS, T79.A11S, T79.A12S, T79.A19S, T79.A21S, T79.A22S, T79.A29S, T79.A3XS, T79.A9XS
Malnutrition/unintended weight loss	E40.*-E43.*, E44.0*, E44.1*, E45.*, E46.*, E64.0*, R62.0*, R62.50-R62.52, R62.59, R63.0*, R63.3*, R63.4*, R63.6*, R64.*
Migraine Headache	G43.*
Osteoarthritis	M15.*-M19.*
Peripheral Artery Disease	E08.52, E09.52, E10.51, E10.52, E10.59, E11.51, E11.52, E11.59, E13.51, E13.52, E13.59,<br>I70.0*, I70.2*-I70.7*, I70.90-I70.932, I73.9*, I77.1*, I96.*, L97.*, I74.10, I74.3, I74.4, I74.5, I74.8, I75.02
Pneumonia	A02.22, A20.2*, A21.2*, A22.1*, A31.0*, A37.01, A37.11, A37.81, A37.91, A42.0*, A43.0*, A48.1*, A78.*, B01.2*, B05.2*, B25.0*, B37.1*, B38.0*, B38.1*, B38.2*, B39.0*, B39.1*, B39.2*, B39.5*, B39.9*, B44.0*, B58.3*, B59.*, B77.81, J12.0*-J12.3*, J12.81, J12.89, J12.9*, J13.*-J15.*,<br>J16.0*, J16.8*, J17.*, J18.0*, J18.1*, J18.8*, J18.9*, J85.0*, J85.1*, J85.2*
Urinary Tract Infection	N39.0*

APPENDIX 5. Medications used for comorbidity adjustment.

**Antihypertensives and Diuretics**	
Angiotensin receptor blocker (ARB)	Azilsartan, candesartan, eprosartan, irbesartan, losartan, Olmesartan, telmisartan, valsartan
Angiotensin-converting enzyme (ACE) inhibitor	Benazepril, captopril, enalapril, fosinopril, lisinopril, moexipril, perindopril, quinapril, ramipril, trandolapril
Calcium channel blocker	Amlodipine, diltiazem, felodipine, isradipine, nicardipine, nisoldipine, verapamil
Beta Blocker	Acebutolol, atenolol, betaxolol, bisoprolol, carvedilol, labetalol, metoprolol, nadolol, nebivolol, penbutolol, pindolol, propranolol, sotolol
Potassium-sparing diuretic	Triamterene, eplerenone, spironolactone, amiloride
Thiazide diuretic	Chlorthalidone, hydrochlorothiazide, indapamide, metolazone
Loop diuretic	Furosemide, bumetanide, torsemide
Alpha antagonists	Doxazosin, prazosin, terazosin
Vasodilators	Hydralazine, minoxidil
**Antithrombotics**	
Anticoagulants	Apixaban, dabigatran, edoxaban, rivaroxaban, warfarin
Antiplatelets	Aspirin, clopidogrel, prasugrel, ticagrelor
**Lipid lowering therapy**	
Statins	Atorvastatin, rosuvastatin, lovastatin, pitavastatin, pravastatin, simvastatin, fluvastatin
Fibrates	Gemfibrozil, fenofibrate, clofibrate
Bile acid sequestrants	Colestipol, cholestyramine resin, colesevelam
Proprotein convertase subtilisin/kexin type 9 (PCSK9) inhibitor	Evolocumab, Alirocumab

Ezetimibe	Ezetimibe
Icosapent ethyl	Omega-3 acid ethyl esters (USP) or icosapent ethyl
**Antiglycemics**	
Metformin	Metformin
Insulin	All insulins
Glucagon-like peptide (GLP)-1 agonists	Semaglutide, liraglutide, dulaglutide, exenatide, lixisenatide
Sodium-Glucose Cotransporter 2 (SGLT2) inhibitors	Empagliflozin, canagliflozin, ertugliflozin, dapagliflozin
Sulfonylureas	Glipizide, glimepiride, glyburide
Dispeptidyl Peptidase IV (DDP-IV) inhibitors	Sitagliptin, saxagliptin, linagliptin, alogliptin
Thiazolidinediones	pioglitazone, rosiglitazone
**Antidepressants, Anxiolytics, and Antipsychotics**	
SSRI	citalopram, escitalopram, fluoxetine, fluvoxamine, paroxetine, sertraline, vilazodone
SNRI	venlafaxine, desvenlafaxine, duloxetine, levomilnacipran, milnacipran
TCA	amitriptyline, amoxapine, clomipramine, desipramine, doxepin, imipramine, nortriptyline, protriptyline, trimipramine
MAOI	isocarboxazid, phenelzine, tranylcypromine, selegiline
Buspirone	Buspirone
Benzodiazepines	alprazolam, chlordiazepoxide, clonazepam, clorazepate, diazepam, estazolam, flurazepam, lorazepam, midazolam, oxazepam, temazepam, triazolam
Trazodone	Trazodone
Bupropion	Bupropion
Mirtazapine	Mirtazapine
GABA analogs	Pregabalin, Gabapentin
First-generation typical antipsychotics	fluphenazine, haloperidol, perphenazine, trifluoperazine, thiothixene, chlorpromazine, thioridazine, loxapine, molindone, mesoridazine

Second-generation antipsychotics	aripiprazole, asenapine, brexpiprazole, cariprazine, clozapine, iloperidone, lurasidone, olanzapine, paliperidone, quetiapine, risperidone, ziprasidone
**Endocrine**	
Thyroid Replacement	levothyroxine, liothyronine, liotrix, desiccated thyroid
Bisphosphonates	alendronate, risedronate, ibandronate, zoledronic acid, etidronate, pamidronate, tiludronate
**Respiratory**	
SABA	albuterol, levalbuterol
SAMA	ipratropium
LABA	salmeterol, formoterol, arformoterol, indacaterol, olodaterol, vilanterol
LAMA	tiotropium, aclidinium, umeclidinium, glycopyrrolate, revefenacin
ICS	beclomethasone, budesonide, ciclesonide, flunisolide, fluticasone propionate, fluticasone furoate, mometasone
**Gastrointestinal**	
Proton pump inhibitors	omeprazole, esomeprazole, lansoprazole, dexlansoprazole, pantoprazole, rabeprazole
Histamine receptor blockers	famotidine, cimetidine, nizatidine
**Opioids**	codeine, hydrocodone, oxycodone, morphine, hydromorphone, oxymorphone, fentanyl, methadone, tramadol, tapentadol, buprenorphine
**Antibiotics**	
Penicillins w/wo Beta-Lactamase inhibitors	penicillin, amoxicillin, ampicillin, oxacillin, nafcillin, dicloxacillin, piperacillin, ticarcillin, amoxicillin-clavulanate, ampicillin-sulbactam, piperacillin-tazobactam, ticarcillin-clavulanate
First Generation Cephalosporins	cephalexin, cefazolin
Second generation cephalosporins	cefuroxime, cefaclor, cefoxitin
Third generation cephalosporins	ceftriaxone, cefotaxime, ceftazidime, cefdinir, cefixime
Fourth generation cephalosporins	Cefepime
Fifth generation cephalosporins	ceftaroline
Carbapenems	imipenem, meropenem, ertapenem, doripenem

Monobactams	Aztreonam
Macrolides	azithromycin, clarithromycin, erythromycin
Fluoroquinolones	ciprofloxacin, levofloxacin, moxifloxacin, ofloxacin, delafloxacin
Tetracyclines	tetracycline, doxycycline, minocycline, demeclocycline, omadacycline, eravacycline
Aminoglycosides	gentamicin, tobramycin, amikacin, streptomycin, plazomicin
Sulfonamides	sulfamethoxazole-trimethoprim (SMX-TMP), sulfadiazine, sulfisoxazole
Lincosamides	Clindamycin
Glycopeptides	vancomycin, telavancin, dalbavancin, oritavancin
Oxazolidinones	linezolid, tedizolid
Nitroimidazoles	metronidazole, tinidazole

APPENDIX 6. Implausible measurement cutoffs.

Measurement	Lower limit	Upper limit
**Laboratory Measures**		
HbA1c (%)	3.0	20.0
HDL Cholesterol (mg/dL)	5	200
LDL Cholesterol (mg/dL)	5	1000
Total Cholesterol (mg/dL)	10	2000
Serum Triglycerides (mg/dL)	5	2000
Serum Creatinine (mg/dL)	0.1	20
AST (U/L)	1	2000
ALT (U/L)	1	2000
White Blood Cell Count (cells/uL)	0.1	100
Hemoglobin (g/dL)	0.5	30
Hematocrit (%)	10	90
Platelet Count (thousands/uL)	1	1000
Potassium	1	15
Sodium	100	255
**Physical Measures**		
SBP (mmHg)	40	300
DBP (mmHg)	40	200
BMI (m/kg^2)	10	100
Temperature (degrees Fahrenheit)	80	115
Pulse Rate (beats per minute)	20	300
Oxygen saturation (%)	50	100
Height (in)	20	100
Weight (pounds)	40	1300
Respiratory rate (respirations per minute)	5	70

DEVIATIONS (pre-modeling)

The home oxygen, wheelchair, and home hospital bed values in the CFI, derived from procedure data, were missing at an extremely high rate, so these adjustment variables were excluded. Very few patients had preceding carotid endarterectomy or cardiac ablation, so these variables were excluded. The CFI definition of heart failure was highly collinear with the Elixhauser definition, so the CFI definition was dropped.

ATTESTATION

I, Jay B. Lusk, the corresponding author for this work and primary analyst, attest that this analysis will be executed faithfully according to the plan detailed above, that any deviations will be disclosed transparently in the final manuscript, and that this plan was committed prior to estimation of the primary study results. This analysis plan was locked on 07/25/2025.

Jay B. Lusk, MD

Digitally signed by Jay B. Lusk, MD
Date: 2025.07.25 12:25:10 -04'00'
