# Supplementary material for: Uptake and safety profile of anti-amyloid immunotherapies in routine clinical practice
Source: J Alzheimers Dis Rep. 2026 Sep 30;10:25424823261483526. doi: 10.1177/25424823261483526 (PMC13662371; doi:10.1177/25424823261483526)

Supplemental Figure 1. Crude and adjusted survival curves. Composite of ICH, Ischemic Stroke, or MI

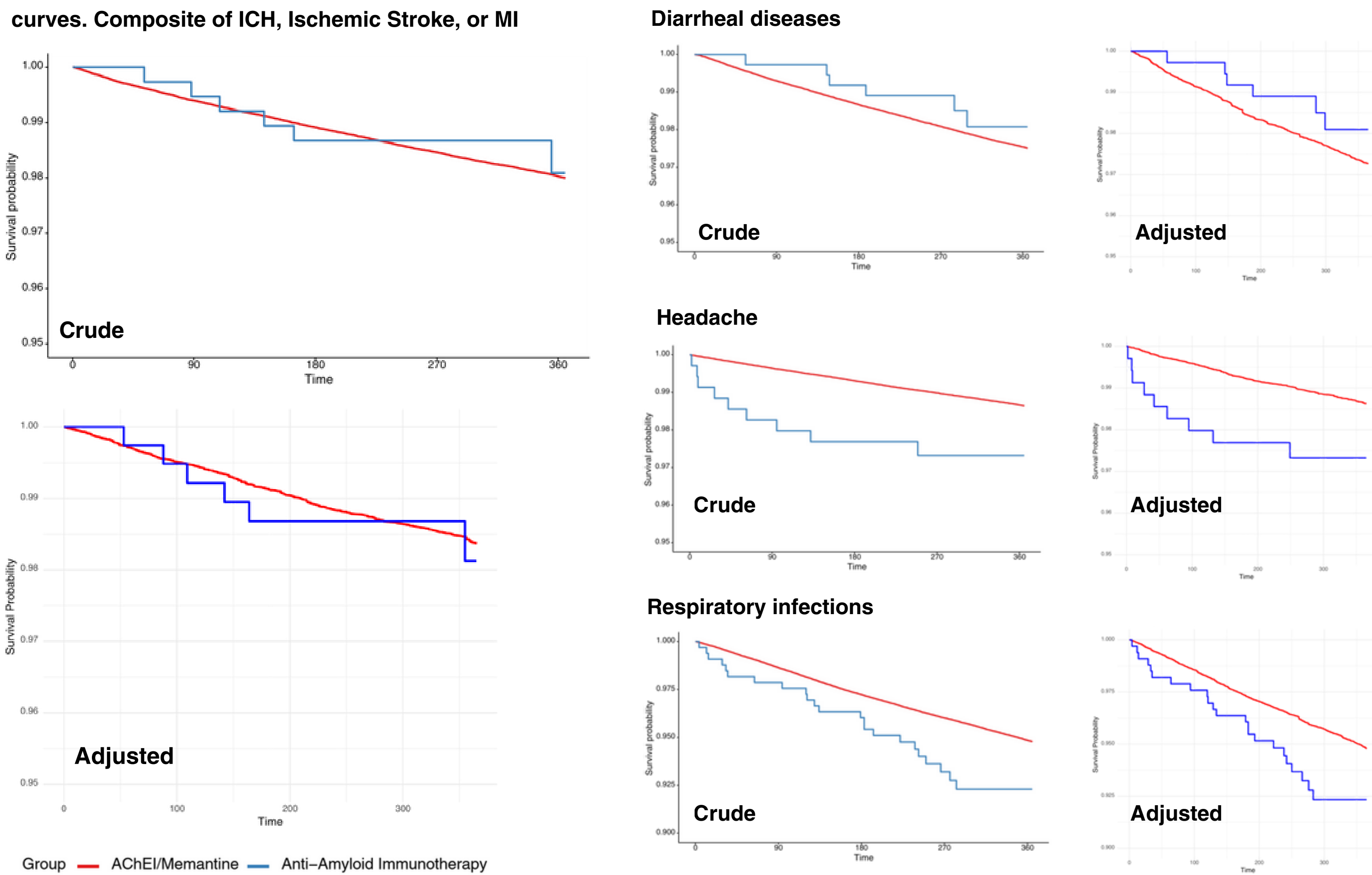

Supplement: Supplementary file 2 [file sj-pdf-3-alr-10.1177_25424823261483526.pdf]
